# An efficient and scalable graph modeling approach for capturing information at different levels in next generation sequencing reads

**DOI:** 10.1186/1471-2105-14-S11-S7

**Published:** 2013-09-13

**Authors:** Julia D Warnke, Hesham H Ali

**Affiliations:** 1 Department of Pathology and Microbiology, University of Nebraska Medical Center, Omaha, NE 68198, USA; 2 College of Information Technology, University of Nebraska at Omaha, Omaha, NE 68182, USA

## Abstract

**Background:**

Next generation sequencing technologies have greatly advanced many research areas of the biomedical sciences through their capability to generate massive amounts of genetic information at unprecedented rates. The advent of next generation sequencing has led to the development of numerous computational tools to analyze and assemble the millions to billions of short sequencing reads produced by these technologies. While these tools filled an important gap, current approaches for storing, processing, and analyzing short read datasets generally have remained simple and lack the complexity needed to efficiently model the produced reads and assemble them correctly.

**Results:**

Previously, we presented an overlap graph coarsening scheme for modeling read overlap relationships on multiple levels. Most current read assembly and analysis approaches use a single graph or set of clusters to represent the relationships among a read dataset. Instead, we use a series of graphs to represent the reads and their overlap relationships across a spectrum of information granularity. At each information level our algorithm is capable of generating clusters of reads from the reduced graph, forming an integrated graph modeling and clustering approach for read analysis and assembly. Previously we applied our algorithm to simulated and real 454 datasets to assess its ability to efficiently model and cluster next generation sequencing data. In this paper we extend our algorithm to large simulated and real Illumina datasets to demonstrate that our algorithm is practical for both sequencing technologies.

**Conclusions:**

Our overlap graph theoretic algorithm is able to model next generation sequencing reads at various levels of granularity through the process of graph coarsening. Additionally, our model allows for efficient representation of the read overlap relationships, is scalable for large datasets, and is practical for both Illumina and 454 sequencing technologies.

## Background

Next generation sequencing has been responsible for numerous advances in the biological sciences allowing for the rapid production of sequencing data at rates not previously possible. Next generation sequencing has allowed for much innovative research in fields such as cancer genomics [1-3], epigenetics [4,5], and metagenomics [6,7]. These instruments are capable of producing several millions to billions of short reads in a single run. These reads are usually a small fraction of the original genome and do not contain much information individually. The massive amount of data that next generation sequencing technologies have produced has necessitated the development of efficient algorithms for short read analysis. Next generation sequencing technologies generate reads at high levels of genome coverage causing many of the reads to overlap. Specialized software programs called assemblers utilize these overlap relationships to organize, order, and align reads to produce long stretches of continuous sequence called contigs, which can be used for downstream analysis. Graph models for providing structure for the reads and their overlap relationships form the foundation of many of these assembly algorithms [8].

Metagenomics is a field of research that focuses on the sequencing of communities of organisms. This adds an additional layer of complexity to the analysis of short reads produced from metagenomics samples containing multiple sources of genetic information. Often these reads must be clustered or binned into their respective genomes before assembly or analysis of the reads can take place to avoid chimeric assembly results [9]. Multiple clustering and binning algorithms have been developed to address this issue [10-12]. While assembly results have been shown to be substantially improved by clustering metagenomics data before sequence assembly [13], overlap relationships retained by the assembly overlap graph are lost, leading to the removal of key global read overlap relationships and read similarities.

To address this issue, we previously introduced a short read analysis algorithm [14] that utilizes an overlap graph to model reads and their overlap relationships. Our algorithm utilizes Heavy Edge Matching (HEM) and graph coarsening methods [15] to efficiently reduce the overlap graph iteratively and to generate clusters of reads. At each graph coarsening iteration the algorithm outputs the reduced graph, producing a series of graphs representing the original read dataset across a spectrum of granularities. In comparison, most previous methods rely on a single graph to represent the read dataset, which may not capture all dataset features. The goal of our research is to create a multilevel approach that will allow for the extraction and analysis of dataset features at different information granularities that can be integrated into the assembly or analysis process. In our previous work, we applied our algorithm to cluster simulated reads representing a metagenomics dataset produced by the 454 technology. We then applied our algorithm to 454 bacterial datasets downloaded from NCBI to test our algorithm's ability to efficiently reduce and store the overlap graph. In this paper, we test the scalability of our algorithm and its ability to accurately cluster simulated Illumina read datasets at different genome coverages. We compare our algorithm's performance when applied to simulated 454 reads versus simulated Illumina reads. We also conduct a study using an Illumina metagenomics dataset downloaded from NCBI to evaluate the scalability of our algorithm. The obtained results show that our algorithm was able to substantially reduce the Illumina metagenomics overlap graph size and is scalable for large datasets. Results also demonstrate that our algorithm is practical for both 454 and Illumina data.

## Results and discussion

In this section, we apply our graph coarsening and clustering algorithm to three Illumina metagenomics read datasets simulated at 5x, 15x, and 25x genome coverage. We evaluate our algorithm's graph coarsening and clustering results and compare them to results obtained by clustering a similar 454 metagenomics read dataset. Finally, we apply our algorithm to a large Illumina metagenomics dataset to demonstrate its scalability and ability to reduce large datasets. For all datasets, we report read overlapping and graph coarsening runtimes when ran on single or multiple compute nodes in a high performance computing environment.

### Metagenomics clustering of simulated Illumina and 454 reads

For this study we generated Illumina read datasets from the eight reference genomes downloaded from NCBI RefSeq [16] used to generate the 454 metagenomics dataset in [14]. These reference genomes were selected at various levels of homology. Half of the bacterial genomes were chosen from the phylum Firmicutes and the remaining half were chosen from the phylum Actinobacteria. Pairs of reference genomes were chosen from the same order.

The software package ART [17] was used to simulate Illumina reads with a read length of 100 bp from each genome at 5x, 15x, and 25x coverage. The characteristics of the 454 metagenomics dataset and the Illumina datasets can be found in table 1 developed in [14] and table 2, respectively.

**Table 1:** Metagenomics 454 read dataset.

Accession #	Organism	Genome Length(bp)	Number of Reads	Avg. Read Length (bp)
NC_012472	*Bacillus cerus*	5 269 628	40 000	445

NC_017138	*Bacillus megaterium*	4 983 975	40 000	440

NC_017999	*Bifidobacterium bifidum*	2 223 664	40 000	406

NC_014656	*Bifidobacterium longum*	2 265 943	40 000	408

NC_017465	*Lactobacillus fermentum*	2 100 449	40 000	467

NC_017486	*Lactobacillus lactis*	2 399 458	40 000	461

NC_011896	*Mycobacterium leprae*	3 268 071	40 000	413

NC_017523	*Mycobacterium tuberculosis*	4 398 812	40 000	420

**Table 2:** Metagenomics Illumina read datasets.

Accession #	Organism	Number of Reads (5x)	Number of Reads (15x)	Number of Reads (25x)
NC_012472	*Bacillus cereus*	263 480	790 440	1 317 400

NC_017138	*Bacillus megaterium*	249 163	747 507	1 245 833

NC_017999	*Bifidobacterium bifidum*	111 180	333 540	555 900

NC_014656	*Bifidobacterium longum*	113 295	339 885	566 475

NC_017465	*Lactobacillus fermentum*	105 008	315 035	525 058

NC_017486	*Lactobacillus lactis*	119 970	359 910	599 850

NC_011896	*Mycobacterium leprae*	163 392	490 168	816 953

NC_017523	*Mycobacterium tuberculosis*	219 940	659 820	1 099 700

Total Reads	■	1 345 428	4 036 305	6 727 169

Eight compute nodes on the commercial strength Firefly cluster located at the Holland Computing Center were used for read overlapping of the simulated Illumina metagenomics dataset [18]. After this was completed, the graph coarsening algorithm was applied to the read overlaps that were output by the overlap algorithm. Graph coarsening was run on a single node on the Firefly computer. The minimum for the ratio of nodes successfully matched to total graph size was .01. The minimum edge density was set to a threshold of 50.

**Figure 1:**
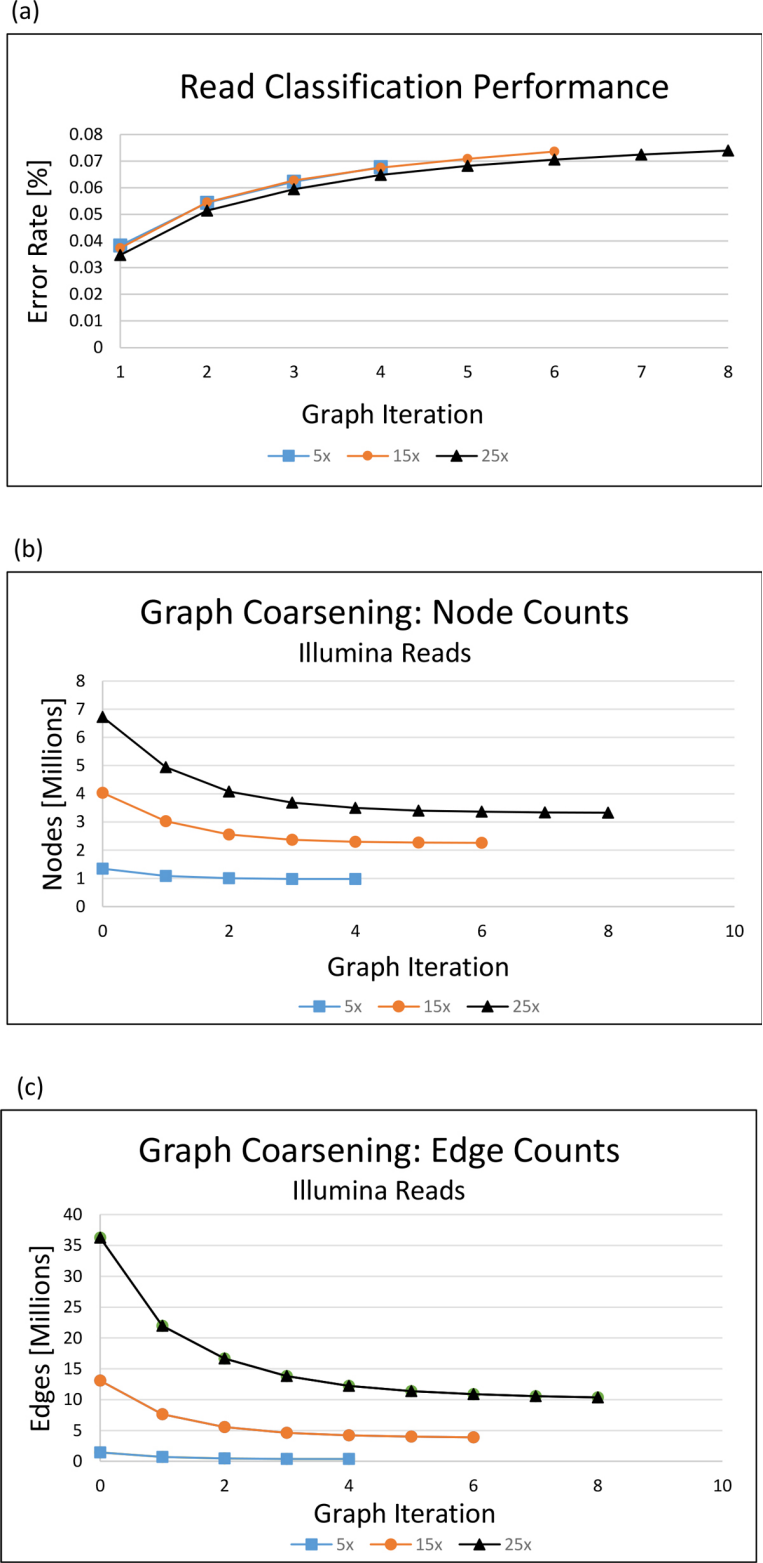
**Clustering quality and graph coarsening statistics for simulated Illumina metagenomics data**. An Illumina metagenomics dataset was simulated from eight bacterial reference genomes downloaded from NCBI. The read classification error rate is shown for each graph coarsening iteration. (b) Node counts. The number of nodes at each graph coarsening iteration (c) Edge counts. The number of edges at each coarsening iteration.

**Figure 2:**
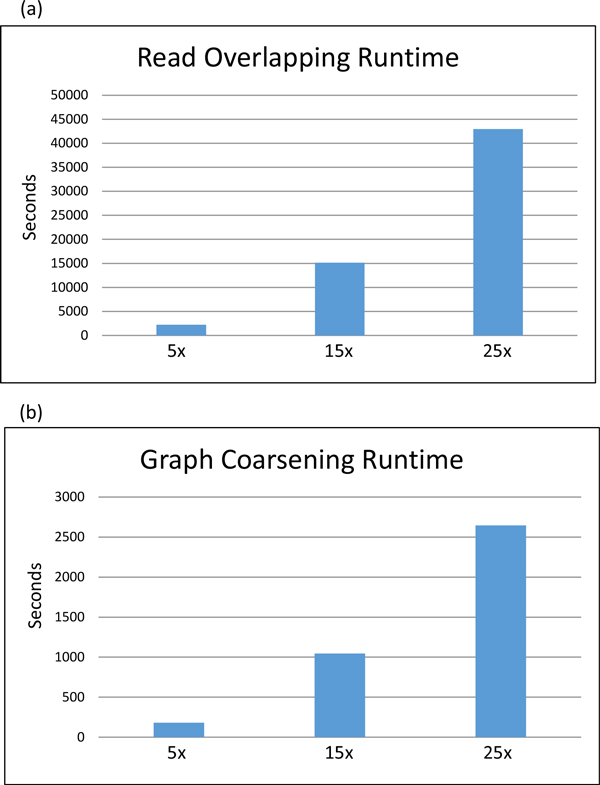
**Read overlapping and graph coarsening runtimes for simulated Illumina metagenomics data**. (a) Read overlapping runtimes for the simulated Illumina metagenomics datasets versus dataset coverage. (b) Graph coarsening runtimes for the simulated Illumina metagenomics datasets versus dataset coverage.

Clusters were generated from the reduced graph at each graph coarsening iteration. We assigned each cluster and its reads a classification at the species level by majority read vote. Our algorithm's cluster classification error rate was defined to be the percentage of misclassified reads. The error rates for the classifications at the species level for various graph coarsening iterations of the simulated Illumina datasets are shown in Figure 1.a. 1.b and 1.c display the node and edge counts for each graph coarsening iteration, respectively. The read overlapping and graph coarsening runtimes can be found in Figure 2.

**Figure 3:**
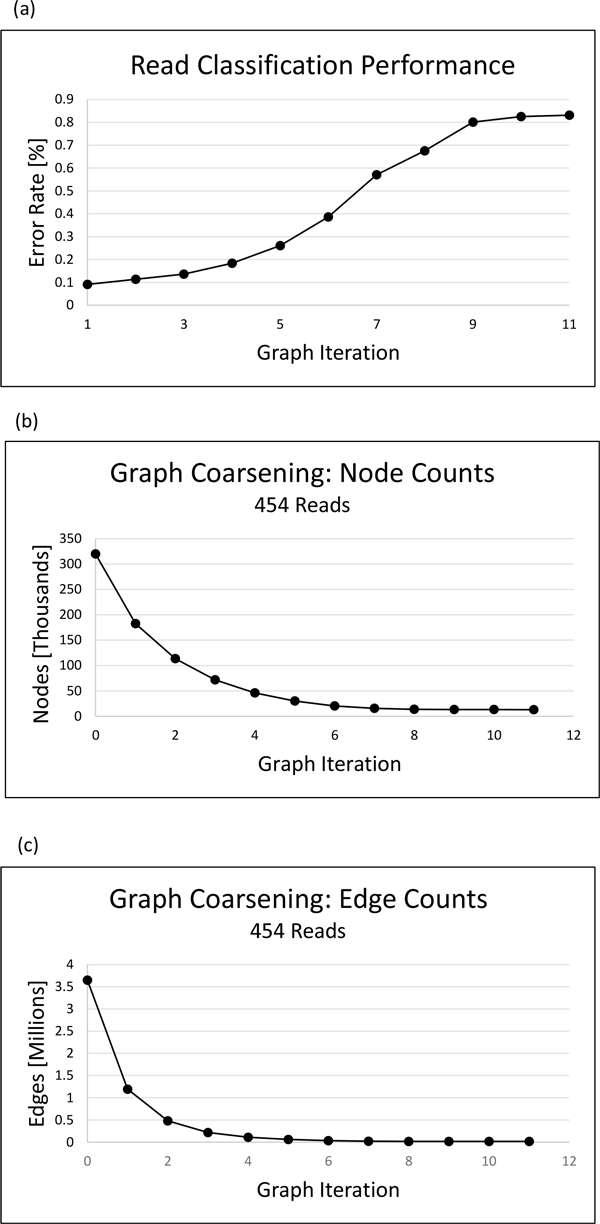
**Clustering quality and graph coarsening statistics for simulated 454 metagenomics data**. (a) Read clustering and classification was performed on a simulated 454 metagenomics dataset generated from eight bacterial genomes. The read classification error rate is shown for each graph coarsening iteration. (b) Node counts. The number of nodes at each graph coarsening iteration (c) Edge counts. The number of edges at each graph coarsening iteration.

For the purpose of comparing our algorithm's performance when applied to datasets generated by different sequencing technologies, we reran our algorithm on the 454 metagenomics dataset in [14]. All runtime parameters remained the same for both the simulated Illumina and 454 read datasets. The error rates for read classification at the species level for each graph coarsening iteration of the simulated 454 read dataset can be found in Figure 3.a.

The node and edge counts can be found in Figure 3.b and 3.c, respectively. Node counts are also recorded in table 3. The graph coarsening algorithm was able to reduce the node count of original overlap graph 1.37 fold, 1.79 fold, and 2.02 fold for the 5x, 15x, and 25x read datasets, respectively, indicating that graph coarsening effectiveness may increase with increasing dataset coverage.

**Table 3:** Node counts per graph coarsening iteration.

Iteration	0	1	2	3	4	5	6
*5x Illumina*	1345428	1088207	1001256	982272	978758	n/a	n/a

*15x Illumina*	4036305	3030374	2551815	2365898	2296455	2270702	2260151

*25x Illumina*	6727169	4941413	4073476	3685025	3494872	3405273	3363256

*454 Reads*	320 000	182 532	113 382	71862	45991	29846	20318

*Bioreactor*	9641139	5419384	4771806	419376	3690376	3248944	2866551

**Iteration**	**7**	**8**	**9**	**10**	**11**	**12**	**13**

*5x Illumina*	n/a	n/a	n/a	n/a	n/a	n/a	n/a

*15x Illumina*	n/a	n/a	n/a	n/a	n/a	n/a	n/a

*25x Illumina*	3342376	3330806	n/a	n/a	n/a	n/a	n/a

*454 Reads*	15592	13747	13215	13071	13026	n/a	n/a

*Bioreactor*	2535701	2248986	2002686	1792359	1613895	1463064	1334821

**Iteration**	**14**	**15**	**16**	**17**	**18**	**19**	**20**

*5x Illumina*	n/a	n/a	n/a	n/a	n/a	n/a	n/a

*15x Illumina*	n/a	n/a	n/a	n/a	n/a	n/a	n/a

*25x Illumina*	n/a	n/a	n/a	n/a	n/a	n/a	n/a

*454 Reads*	n/a	n/a	n/a	n/a	n/a	n/a	n/a

*Bioreactor*	*1226384*	1134971	1058853	996532	94591	907599	877863

**Iteration**	**21**	**22**	**23**	**24**	**25**	**26**	**27**

*5x Illumina*	n/a	n/a	n/a	n/a	n/a	n/a	n/a

*15x Illumina*	n/a	n/a	n/a	n/a	n/a	n/a	n/a

*25x Illumina*	n/a	n/a	n/a	n/a	n/a	n/a	n/a

*454 Reads*	n/a	n/a	n/a	n/a	n/a	n/a	n/a

*Bioreactor*	*855983*	840179	828999	821065	815434	811363	n/a

The results from the graph coarsening and clustering of the simulated Illumina and 454 read dataset demonstrate that the read error rates for the two sequencing technologies are comparable and that our algorithm can be successfully applied to both 454 and Illumina reads. Read overlapping and graph coarsening runtimes demonstrate the scalability of the algorithm for datasets of increasing size and genome coverage. For the largest simulated Illumina dataset, read overlapping was completed on eight nodes in less than twelve hours. Graph coarsening was completed on a single node in less than forty-five minutes for the largest simulated Illumina dataset. The read overlapping and graph coarsening runtimes for the remaining datasets can be found in tables 4 and 5, respectively.

**Table 4:** Read overlapping runtime (8 Nodes).

Dataset	Runtime (seconds)
*5x Illumina*	2231

*15x Illumina*	15 130

*25x Illumina*	42 973

*454 Reads*	3054

*Illumina bioreactor* *metagenomics* *(30 Nodes)*	26 529

**Table 5:** Graph coarsening runtime (serial merge sort).

Dataset	Runtime (seconds)
*5x Illumina*	181

*15x Illumina*	1045

*25x Illumina*	2646

*454 Reads*	390

### Illumina bioreactor metagenomics dataset

The results from the simulated metagenomics study demonstrated the algorithm's ability to reveal incremental levels of information in read datasets and that it can be extended to both 454 and Illumina read datasets. However, for this algorithm to be practical for a wide range of sequencing applications, we must demonstrate the scalability of this algorithm for large read datasets. For this purpose, we applied our algorithm to a large Illumina bioreactor metagenomics dataset and evaluated its runtime and ability to reduce a large overlap graph.

We downloaded an Illumina bioreactor metagenomics dataset from the NCBI sequence read archive [19]. Table 2 describes the characteristics of this dataset. Paired-end reads were split for a total of 9,641,139 single reads. Any low quality read ends were trimmed with a minimum quality score of twenty using the FASTQ Quality Trimmer of the FASTX-toolkit [20].

**Figure 4:**
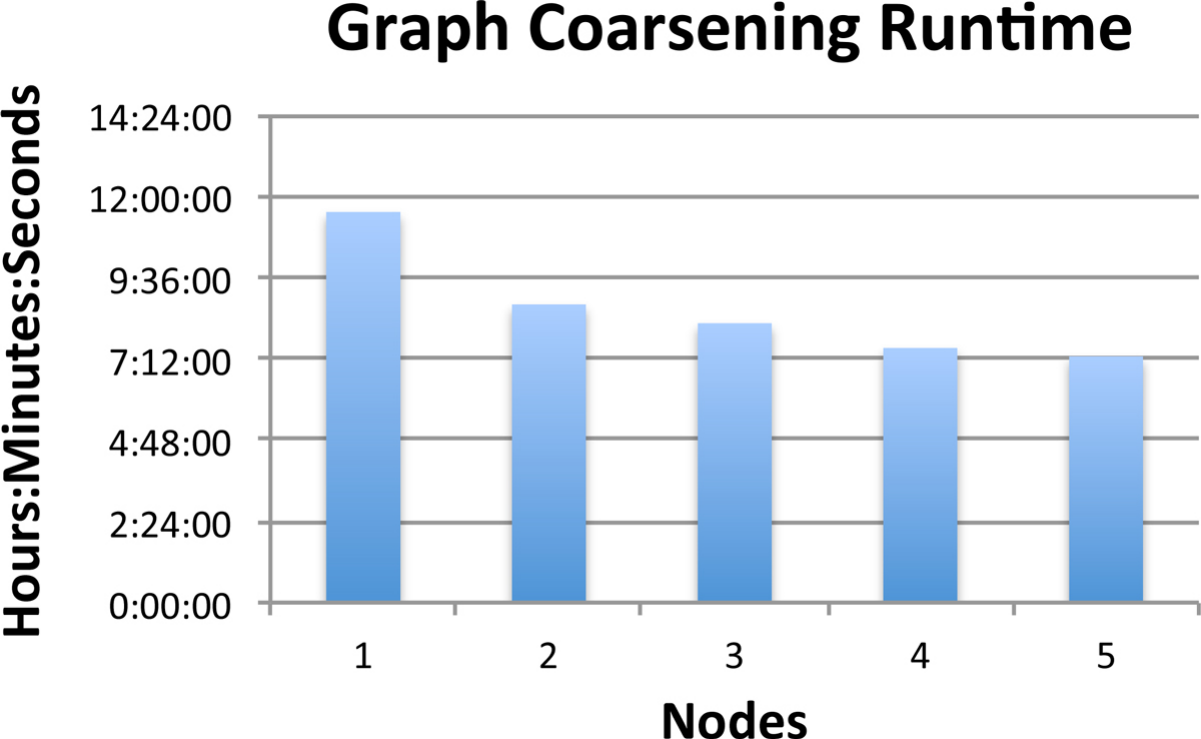
**Graph coarsening runtimes: Illumina bioreactor metagenomics data**. The runtime for the graph coarsening algorithm applied to the Illumina bioreactor metagenomics dataset downloaded from NCBI's sequence read archive. The graph coarsening algorithm was run multiple times with one to five compute nodes utilized in the parallel merge sort step.

**Figure 5:**
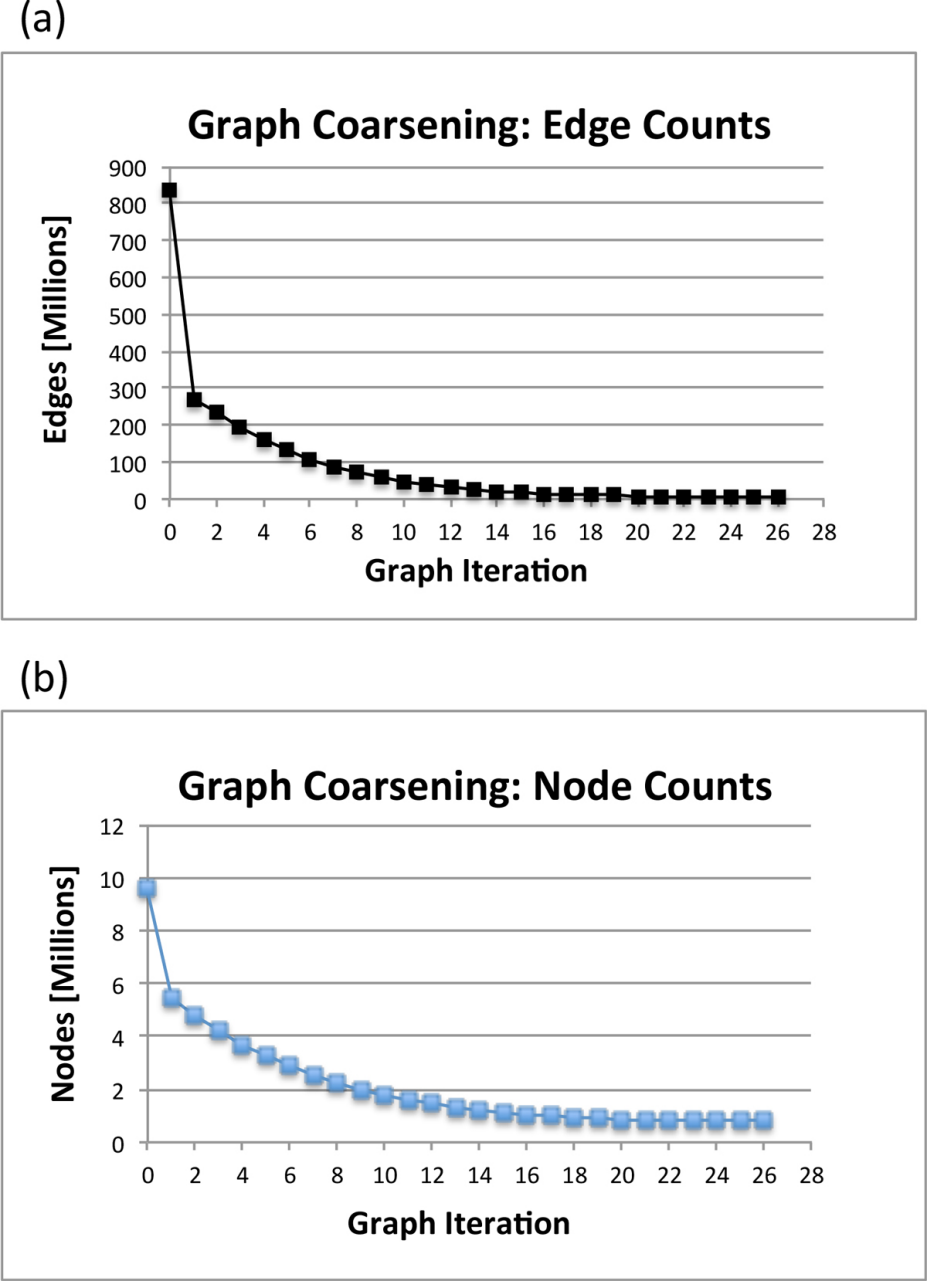
**Graph coarsening statistics: Illumina bioreactor metagenomics data**. (a) The number of edges is shown for each graph coarsening iteration. (b) The number of nodes is shown for each graph coarsening iteration.

Read overlapping was completed on thirty nodes of the Firefly computing cluster [18]. We then applied the graph coarsening algorithm to the overlap relationships produced by the read overlapper. We applied our graph coarsening algorithm multiple times to the dataset with varying numbers of compute nodes added to the parallel merge sort algorithm. The minimum for the ratio of nodes successfully matched to graph size was .01. The minimum edge density threshold was set to 50. The node counts for each graph coarsening iteration are recorded in table 3. The runtime for the read overlapping of the Illumina bioreactor dataset is shown in table 4. Figure 4 shows the runtime for the graph coarsening algorithm versus the number of compute nodes added to the parallel merge sort algorithm. The results demonstrate that our algorithm is scalable for larger metagenomics datasets. Figure 5 displays the reduction in node and edge counts per graph coarsening iteration. The number of edges was reduced approximately 168 times from the original overlap graph by the twenty-sixth graph coarsening iteration. The number of nodes was reduced approximately twelve times from the original overlap graph by the twenty-sixth graph coarsening iteration. The greatest reduction in edge counts occurred in the first ten graph coarsening iterations and the greatest reduction in node counts occurred in the first fourteen graph coarsening iterations.

## Conclusions

In this paper, we introduced a graph coarsening and clustering algorithm that is able to model reads at multiple levels across a spectrum of information granularities. We demonstrated our algorithm's ability to cluster simulated Illumina metagenomics data at different levels of genome coverage. Clustering error rates of the algorithm applied to simulated Illumina metagenomics reads are comparable to error rates for clustering a simulated 454 metagenomics dataset with similar dataset characteristics. This suggests that our algorithm can be successfully applied to both Illumina and 454 read data. Algorithm runtimes were practical for all datasets. The largest simulated Illumina read dataset required less than twelve hours to complete read overlapping on eight compute nodes. Graph coarsening was completed on a single node in less than forty-five minutes. Read overlapping of the simulated 454 read dataset required less than an hour on eight compute nodes. Graph coarsening required less than seven minutes on a single compute node. The graph coarsening algorithm was more effectively able to reduce the higher coverage simulated Illumina datasets than the lower coverage Illumina datasets.

Finally, we applied our algorithm to a large Illumina metagenomics dataset to demonstrate its scalability. By utilizing parallel computing, our read overlapping algorithm was able to complete less than eight hours. The graph coarsening algorithm completed within approximately seven to twelve hours depending on the number of nodes added to the parallel merge sort portion of the graph coarsening algorithm. The algorithm was able to reduce the number of edges in the overlap graph nearly 168 fold while recording each graph level on disk, allowing a researcher to access the overlap graph at various levels of complexity. We plan to expand our graph coarsening algorithm to a full sequence assembler which will be contrasted to currently available assembly methods. We also plan to conduct further in-depth studies on the impact of input parameters on the graph coarsening process. Most current approaches rely on one overlap graph to capture a single snapshot of the reads and their overlap relationships. In contrast, our proposed assembly algorithm will rely on a series of coarsened graphs to capture both local and global dataset features.

The goal of our research is to develop an analysis method that will allow us to extract features of the read dataset at multiple information granularities to incorporate into the read analysis and assembly process. In the future, we plan to configure the algorithm such that clusters or nodes can be selected at different levels of information granularity. For example, if a node in a reduced graph is over-collapsed, we can select its child nodes from the previous graph iteration instead. We can continue with this zooming-in and zooming-out process, selecting child nodes from previous graph iterations until the desired node criteria is achieved or optimized. This will facilitate a customizable, intelligent approach to the read analysis and assembly problem.

## Methods

### Read overlapping

**Figure 6:**
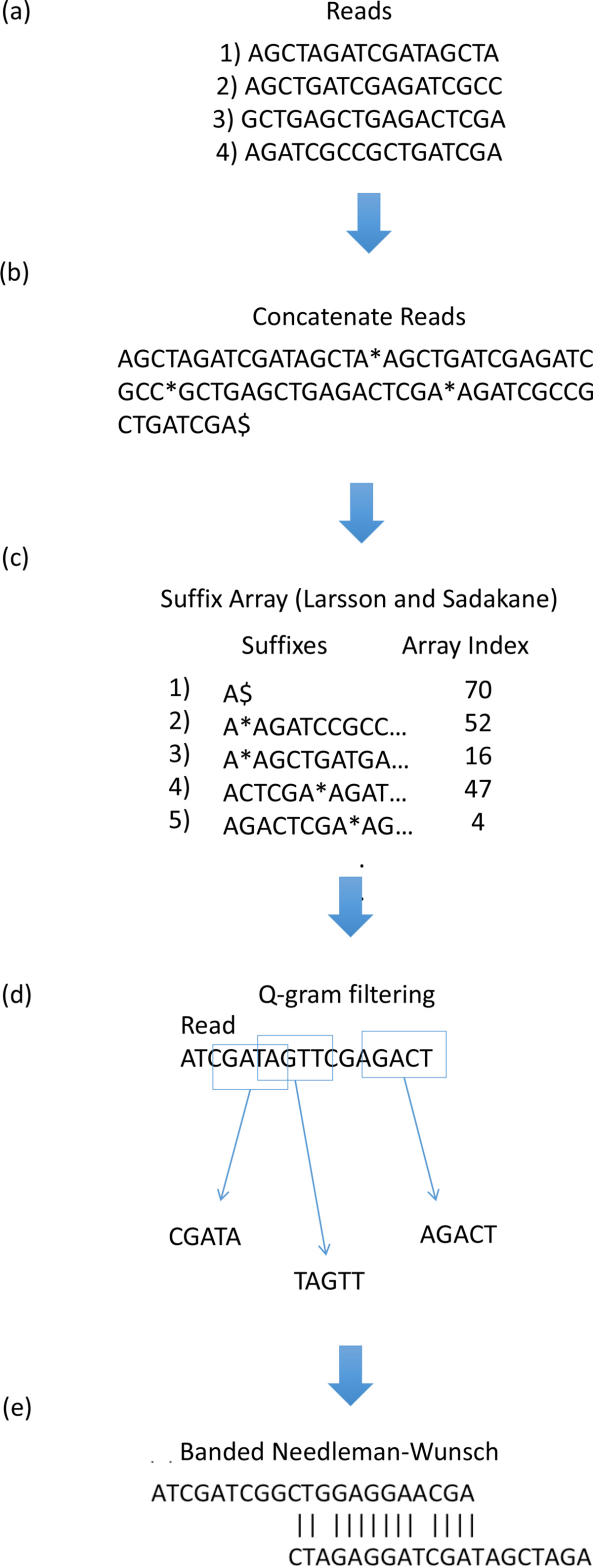
**Read overlapping**. (a) Read dataset (b) Dataset concatenation (c) Suffix array indexing (d) K-mer search and q-gram filtering (e) Banded Needleman-Wunsch alignment.

We have developed an exact match read overlapper for discovering read overlap relationships. All reads are concatenated into a single string and indexed by a suffix array data structure [21]. In succession, each read is split into its *l*-k-1 component k-mer seeds, where *l *is the length of the read. The read overlapper searches the suffix array for exact matches to the k-mer seeds. If an exact match is found, the reference read that contains the hit is selected for comparison to the query read. The reference and query reads are subject to a q-gram filter [22]. If the reads pass the filter, then the hit is extended into a full alignment using a banded Needle-Wunsch algorithm [23]. The alignment length and identity score are used to evaluate the quality of the produced overlap relationship. If the overlap does not meet minimum user-thresholds, then it is excluded from the final read overlap set. The ids of the query and reference reads are recorded along with the alignment length and identity for each overlap that meets minimum threshold requirements. After the completion of the read overlapping process, the overlap set is ordered by parallel merge sort into an edge list. The query and reference read ids become, respectively, the labels of the source and destination nodes of the edges in the overlap graph. Edges that have the same source node are grouped into edge sets within the edge list. Each edge set is sorted by overlap length, from longest overlap length to smallest. The edge list is then indexed by a graph data structure. To address computational requirements for large datasets, we split the read dataset into subsets which are indexed by suffix arrays and sent to compute nodes in pairs to be ran in parallel in high performance computing environments. Figure 6 gives a flow diagram for the read overlapping process.

### Graph theoretic model

Graph theory has become an important tool in many areas of bioinformatics research. The overlap graph forms the structural foundation of our read analysis and clustering algorithm. For this graph theoretic model, there is a one-to-one correspondence between the reads in the read dataset and nodes in the overlap graph. Edges connecting nodes in the overlap graph represent overlap relationships between reads. Each edge stores its corresponding overlap's alignment length and identity score. The overlap graph shares many similarities with the interval graph. The interval graph is one of the perfect graphs in graph theory and has many defined properties, making it a robust model for many applications [24].

**Figure 7:**
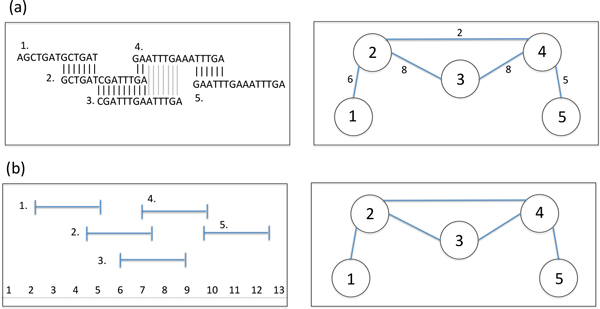
**Graph theoretic model**. (a) The overlap graph. There is a one-to-one correspondence between the nodes in the overlap graph and reads in the read dataset. Edges represent overlap relationships between nodes and store information about the overlap's alignment length and identity. (b) The interval graph. The nodes of the interval graph represent a set of intervals on the real line. Overlaps between intervals are represented by edges.

An example of the overlap graph and interval graph developed in [14] can be found in Figure 7. We use succinct dictionary data structures [25] to index the nodes and edges of the overlap graph in a highly efficient manner allowing us to store our graph in O(*n*) + O(*m*) + 64*m *bits, where *n *and *m *are the total nodes and edges in the graph, respectively. More details on the structure and efficiency of the graph data structures can be found in [14].

### Graph coarsening

We apply Heavy Edge Matching (HEM) to produce a series of coarsened graphs [15]. Graph coarsening provides levels of information about the structure of a graph over a spectrum of graph granularities. A very simple overview of global graph features exists at the coarsest graph levels, while more complex graph details are retained in earlier coarsening iterations and the original overlap graph.

**Figure 8:**
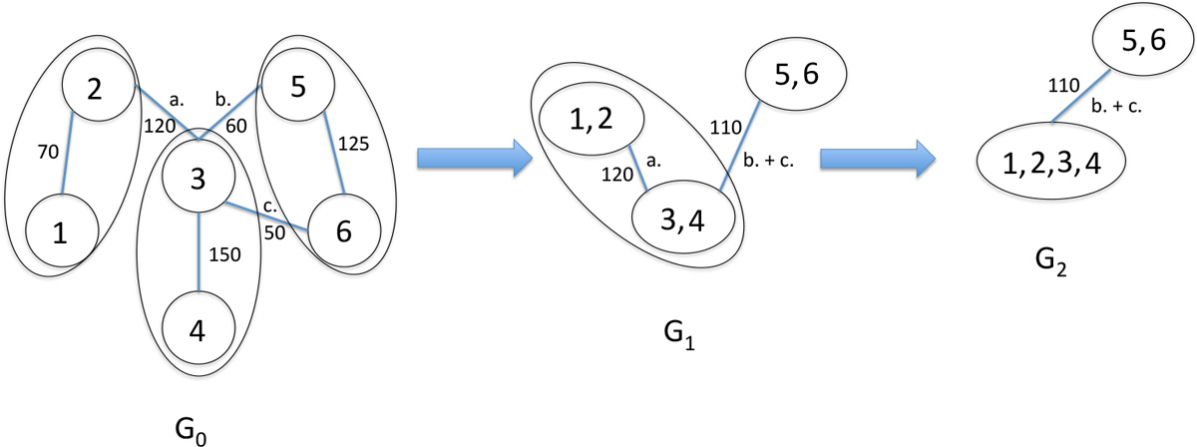
**Graph coarsening**. A maximal matching of size three on G_0_. Each edge in the matching is removed and its endpoints are merged into a single supernode in a new coarsened graph G_1_. Edges b. and c. have the same supernode endpoints and are merged into a single edge. The sum of the edge weights for b and c is assigned to the merged edge. Graph coarsening is repeated on G_1 _to produce G_2_.

Here we provide a description of our HEM algorithm for graph coarsening. Our graph coarsening algorithm attempts to find a maximal matching, a maximal set of edges without common endpoints, over a graph while maximizing for edge weight. The endpoints of these edges are then merged to form supernodes in a new coarsened graph. The steps of our algorithm are as follows. Given an overlap graph G_n _= (V_n_, E_n_), the graph coarsening algorithm visits its nodes in succession over a user-specified number of passes, with the nodes with the largest edge weights visited randomly first followed by nodes with smaller edge weights. In the initial overlap graph, the edge weight is set to the alignment overlap length. We desire to visit nodes with larger overlap lengths first because the larger the overlap shared between two reads, the greater the likelihood that the reads are adjacent to one another in the original target sequence. The edges of the currently selected node *u *are visited from largest edge weight to smallest. The selected node *u *is matched to its first available neighbor *v *and the search through its edges is terminated. If no such neighbor exists or if the edge overlap length falls below a user-provided minimum during the edge search, then *u *remains unmatched. After the matching process is complete, the endpoints of each edge (*u, v*) in the matching are merged into a single supernode *z *in a new coarsened graph, G_n+1_. Each unmatched node is mapped to a new node in the coarsened graph. Each edge from the original graph G_n _is mapped to a new edge in the coarsened graph G_n+1_. The endpoints of an edge in G_n+1 _are the supernodes that contain the endpoints of its corresponding edge in G_n _as component nodes. If two edges in the coarsened graph share the same supernode endpoints, then the edges are merged into a single edge and their weights are summed to form a new edge weight. An example of graph coarsening via HEM can be found in Figure 8 developed in [14].

This graph coarsening process can be applied to the newly coarsened graph G_n+1 _to produce an even coarser graph G_n+2_. This iterative process of node matching and merging produces a series of coarsened graphs G_0_, G_1_, G_2 _... G_n_, where N(|G_0_|) ≥ N(|G_1_|) ≥ N(|G_2_|) ... ≥ N(|G_n_|) representing the dataset across a spectrum of information levels. Graph coarsening is terminated when the ratio of the number of nodes successfully matched to graph size falls below a user minimum.

Four array data structures are used to hold critical information describing the graph coarsening process. For each graph G_0_, G_1_, G_2 _... G_n _in the series of coarsened graphs, there are two arrays, *node_weights *and *edge_weights*. For each supernode *z *in a graph G_n_, *node_weights_n _*records the number of child nodes descended from *z *in G_0 _and *edge_weights_n _*records the total weight of the edges induced by the child nodes. Let *z *be a supernode in a graph G_n+1 _and *u *and *v *be its child nodes in G_n_. We use these arrays to calculate node density with the following equation.

edge_density(u,v) = edge_density(z) =

$$\frac{2*\left(ew\left[u\right]+ew\left[v\right]+w\left(u,v\right)\right)}{\left(nw\left[u\right]+nw\left[v\right]\right)*\left(\left(nw\left[u\right]+nw\left[v\right]\right)-1\right)}$$

where *ew*[u] = *edge_weights_n_*[*u*] and *nw*[u] = *node_weights_n_*[u].

During the matching process, a node *u *will be matched to its neighbor *v *only if *edge_density(u, v*) is greater than a user-provided minimum.

Each graph is also assigned two additional arrays, *node_map *and *node_map_inverse*. For each node *u *in a graph G_n_, *node_map_n_*, records the label of the supernode that *u *is mapped to in G_n+1_. For each supernode *z *in a graph G_n+1_, *node_map_inverse_(n+1) _*records the labels of its child nodes in G_n_. Let *u *and *v *be nodes in G_n _that are mapped to supernode *z *in G_n+1_, then *node_map_n_*[*u*] = *node_map_n_*[*v*] = *z*. If *z *is a supernode in G_n+1_, then *node_map_inverse*_(n*+1*)_[2*z] *= u *and *node_map_inverse*_(n+1)_[2*z+1] *= v*, where (*u, v*) is an edge in a matching M applied to G_n_. If *z *only has one child node *u*, then *node_map_inverse*_(*n+1*)_[2*z] *= u *and *node_map_inverse*_(n+1_)[2*z+1] *= -1*.

**Figure 9:**
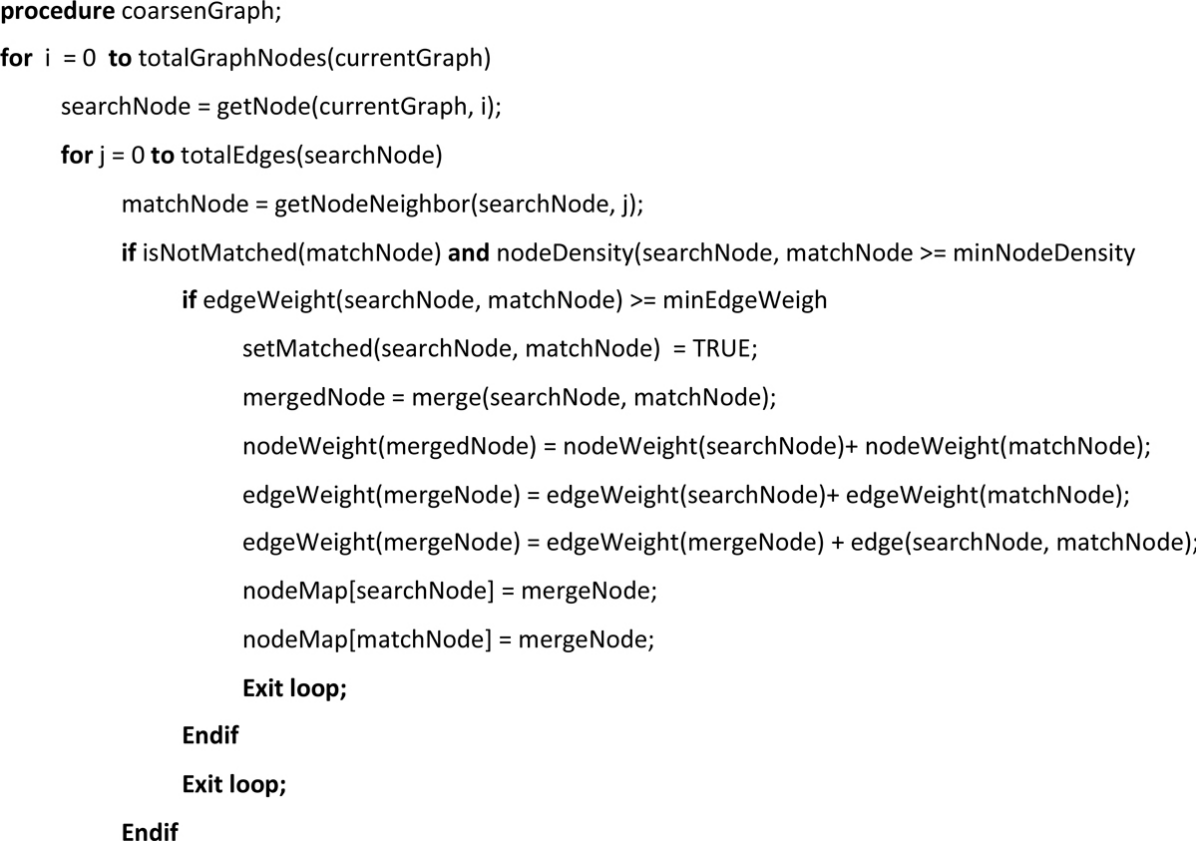
**Pseudocode**. Pseudocode is shown for the graph coarsening procedure.

After the completion of matching, the edges of G_n+1 _are generated from the edges of G_n_. The algorithm labels each edge *e *= (*u, v*) in G_n _to form a new edge *e*_new _= (*node_map_n_*[*u*], node*_map_n_*[*v*]) in G_n+1_. If *node_map_n_*[*u*] = *node_map_n_*[*v*], then *e*_new _is not included in the edge set. The edges are ordered into an edge list by parallel merge sort such that edges with the same source node are grouped into edge sets within the edge list. The edge sets are ordered by descending edge weight. Any edges with the same endpoints in G_n+1 _are merged and their weights are summed. The new edge set is indexed by graph data structures to form G_n+1_. Pseudocode for the graph coarsening process is shown in Figure 9.

### Read cluster generation

Our algorithm uses the *node_map_inverse *arrays to recover reads clusters from a given coarsened graph G_n_. Recall that if *z *is a supernode in G_n_, then *node_map_inverse_n_*[2*z] *= u *and *node_map_inverse_n_*[2*z+1] *= v*, where *u *and *v *are child nodes of *z *in G_n-1_. The labels of the child nodes of *u *in G_n-2 _would therefore be given by *node_map_inverse_(n-1)_*[2*node_map_inverse_n_[2*z] ] and *node_map_inverse_(n-1)_*[2*node_map_inverse_n_[2*z] +1]. The child nodes of *v *would be given by *node_map_inverse_(n-1)_*[2*node_map_inverse_n_[2*z+1] ] and *node_map_inverse*[2*node_map_inverse_n_[2*z+1] +1]. This nested iteration through the *node_map_inverse *arrays continues until the all of the labels of the child nodes of *z *in G_0 _are recovered. Since the label of each node in G_0 _is the id of the read to which it corresponds to in the read dataset, we can use the child node labels to generate the read cluster belonging to the supernode *z*.

### Edge relabeling

Graph traversal of the reduced overlap graph is used to determine an ordering of the nodes in the original, full overlap graph. The end points of each edge in the original overlap graph are relabeled according to the node ordering recovered from the reduced overlap graph. The goal of the edge relabeling process is to organize the edges within the original overlap graph such that many of the edges with common endpoints are close to one another in the graph data structure, facilitating more efficient access to overlap graph information. More details on the reduced graph traversal and edge relabeling process and experiments examining its effectiveness can be found in [14].

## Competing interests

The authors declare that they have no competing interests.

## Authors' contributions

JDW and HHA contributed equally to the novel ideas and introduced concepts of the paper. The algorithms of the paper were designed by both authors and implemented by JDW. The paper was written primarily by JDW, and reviewed and edited by HHA.

## Declarations

The publication costs for this article were funded by the corresponding author.

This article has been published as part of *BMC Bioinformatics *Volume 14 Supplement 11, 2013: Selected articles from The Second Workshop on Data Mining of Next-Generation Sequencing in conjunction with the 2012 IEEE International Conference on Bioinformatics and Biomedicine. The full contents of the supplement are available online at http://www.biomedcentral.com/bmcbioinformatics/supplements/14/S11.
